# Training Load Measures and Biomarker Responses during a 7-Day Training Camp in Young Cyclists—A Pilot Study

**DOI:** 10.3390/medicina57070673

**Published:** 2021-06-29

**Authors:** Yvonne Wahl, Silvia Achtzehn, Daniela Schäfer Olstad, Joachim Mester, Patrick Wahl

**Affiliations:** 1 Institute of Exercise Training and Sport Informatics, German Sport University Cologne, 50933 Cologne, Germany; y.wahl@dshs-koeln.de; 2 The German Research Centre of Elite Sport, German Sport University Cologne, 50933 Cologne, Germany; achtzehn@dshs-koeln.de (S.A.); mester@dshs-koeln.de (J.M.); 3 Polar Electro Oy, 90440 Kempele, Finland; daniela.olstad@polar.com; 4 Institute of Interdisciplinary Exercise Science and Sports Medicine, Medical School Hamburg, 20457 Hamburg, Germany

**Keywords:** biomarker, point-of-care testing, training load, young cyclists

## Abstract

*Background and Objectives*: During intense training periods, there is a high need to monitor the external and especially the internal training load in order to fine-tune the training process and to avoid overreaching or overtraining. However, data on stress reactions, especially of biomarkers, to high training loads in children and youth are rare. Therefore, in this study, we aimed to investigate the training load of youth athletes during a training camp using a multilevel approach. *Materials and Methods*: Six trained youth male cyclists performed a 7-day preseason training camp. To investigate the internal training load, every morning, minimally invasive “point-of-care testing” (POCT) devices were used to analyze the following biomarkers: creatine kinase (CK), blood urea nitrogen (BUN), albumin (Alb), bilirubin (Bil), alanine aminotransferase (ALT), aspartate aminotransferase (AST), and total protein (TP). Additionally, data of training load measures (HR: heart rate, RPE: rating of perceived exertion, sRPE: session-RPE, TRIMP: training impulse, intensity (RPE:HR), and load (sRPE:TRIMP) ratios), self-perception (person’s perceived physical state, questionnaires on muscle soreness, and sleep quality), and measures of the autonomic nervous system (resting heart rate, heart rate variability) were collected. Two days before and after the training camp, subjects performed performance tests (Graded Exercise Test, Wingate Anaerobic Test, Counter Movement Jump). *Results*: Primarily, the biomarkers CK, BUN, and Alb, as well as the self-perception showed moderate to large load-dependent reactions during the 7-day training camp. The biomarkers returned to baseline values two days after the last training session. Power output at lactate threshold showed a small increase, and no changes were found for other performance parameters. *Conclusions*: The study suggests that a multilevel approach is suitable to quantify the internal training load and that different parameters can be used to control the training process. The biomarkers CK, BUN, and Alb are suitable for objectively quantifying the internal training load. The self-perception provides additional subjective information about the internal training load.

## 1. Introduction

The increase in the professionalization of competitive youth sports is associated with an increase in the training load, in terms of exercise intensity and volume. Specialization in high-performance sports starts early currently and children may be at risk of overload subsequently affecting their development. As recently stated by the International Olympic Committee consensus statements, load management is crucial for athletes [1,2]. Especially in training camps, the load is increased in a short time span with a potential risk of overloading [3]. However, little is known about the athletes’ tolerance to such training camps. Therefore, there is a high need to monitor and examine the external training load on the one hand, but on the other hand, and even more important, to investigate the internal training load. Due to the different time courses of physiological, biochemical, performance, and psychological reactions in response to periods of high training load, monitoring of the external and internal training load should be performed in a multilevel approach [4].

The external training load, defined as the work completed by an athlete, can be determined simply by the measurement of speed, distance, altitude difference, and power output [5]. However, especially in group training where often the same external training load is performed by all athletes, the internal training load of each individual athlete to a training stimulus might differ extensively. Therefore, the internal training load should be measured using different methods. Easy measures include the mean heart rate (HR), rating of perceived exertion (RPE), session RPE (s-RPE) [6], training impulse (TRIMP) [7], measurements of performance, psychological questionnaires [8], the assessment of the autonomous nervous system balance (HR and heart rate variability (HRV)) [3,9,10], and sleep [5]. Furthermore, Sanders et al. suggested that ratios between perceptual and physiological indicators of intensity (RPE:HR) and load (sRPE:TRIMP) can provide additional information to monitor the fatigue state of athletes [11]. More complex measures for monitoring the training load and the training status include biomarkers. 

Biomarkers are attractive parameters for quantifying exercise-induced stress and fatigue in different tissues, as each biomarker has a clear physiological link to the tissue being stressed [12]. To study the internal load of different tissues, it is therefore important to analyze a whole set of blood markers. In terms of endurance exercise, the load status of different tissues might be of interest, which can be investigated by the following biomarkers: Muscle damage markers such as creatine kinase (CK) can be released from muscle tissue due to both strenuous mechanical and metabolic exercise [13]. After endurance exercise, peak CK values will be reached after 24 h, remaining elevated up to 48 h or even longer, if athletes continue to train [14,15,16]. Alanine aminotransferase (ALT) and aspartate aminotransferase (AST) are mainly found in the liver, but they are also found in smaller amounts in muscle tissue [17]. AST is immediately released from activated muscles during physical exercise, remaining increased up to 24 h due to muscular damage [14,17,18]. Augmentation of AST and ALT is linked to performance intensity and duration, as found in ultraendurance events [19]. Metabolic markers such as bilirubin (BIL) are influenced by erythrocyte destruction (hemolysis), which was shown to be increased in athletes [14]. The principal source of increased red blood cell turnover is the intravascular hemolysis that is caused by impact with the ground (footstrike hemolysis), mechanical damage to red blood cells during continuous muscle contractions or continuous exposure to high-oxygen flux, causing oxidative damage [14]. Blood urea nitrogen (BUN), a terminal breakdown product of protein, is a classic fatigue marker in endurance disciplines and is elevated by prolonged exercise [20]. The most frequently given reason for increased BUN values in elite athletes is a high training volume associated with a marked increase in energy expenditure, depletion of carbohydrate stores, and subsequent protein breakdown for gluconeogenesis and ATP production [21]. Changes in the concentration of total protein (TP) and albumin (Alb) can be used as parameters for an increased catabolic state after prolonged exercise [22]. TP is a complex mixture of two major groups of proteins, Alb (60% of TP) and globulin. The level of TP in the blood is normally a relatively stable value, reflecting a balance in the loss of old proteins and the production of new proteins. However, TP can increase due to strenuous exercise, dehydration, and inflammation processes [23]. Alb is mainly found in the liver and responsible for 75% of the oncotic pressure of plasma, meaning Alb is mainly responsible to keep water inside the blood vessels. Alb typically decreases during inflammatory processes, when the liver focuses on producing inflammatory proteins. In a few cases, severe protein–energy malnutrition may lead to decreased serum albumin levels as well. 

The former invasive collection of the biomarkers mentioned made their frequent use in young or elite athletes difficult, as the daily training routine should be affected as little as possible by these measures. In contrast, the use of point-of-care testing (POCT) currently allows minimal-invasive measurements daily. Only small amounts of capillary blood from the earlobe or the fingertip are needed, and the results of these measurements are directly available, offering the possibility to directly intervene in the training process. Therefore, such measurements can even be made in a sport-specific field condition, such as training camps [24]. 

To date, there are no studies in young athletes that monitor the external and internal training load using a multilevel approach during periods of high training load. Therefore, the present study aimed to investigate the external and internal training load using biomarkers analyzed with minimally invasive POCT devices, simple training load measures (sRPE, HR, TRIMP), intensity (RPE:HR) and load (sRPE:TRIMP) ratios, psychological questionnaires, HRV (RMSSD: square root of the mean of the sum of the squares of differences; meanRR: mean R-R intervals; SDNN: standard deviation of normal R-R intervals), and performance tests in young cyclists during a 7-day training camp.

## 2. Materials and Methods

### 2.1. Subjects

Six trained male youth cyclists (mean ± SD; age: 15 ± 1 yrs; size: 177.2 ± 8.8 cm; body mass: 58.5 ± 6.9 kg; relative peak oxygen uptake (VO_2_peak): 57.8 ± 4.5 mL·kg^−1^·min^−1^) performed a 7-day training camp (517 km distance, 5820 m of altitude difference). The normal athletes’ weekly training load comprised of 150–200 km and consisted of continuous low-intensity cycling as well as high-intensity interval sessions. The criteria for inclusion, assessed by questionnaire, were no history of mental or physical impairment and no use of any pharmaceutical substances. The study protocol was approved by the Ethics Committee of the German Sport University Cologne (approval code: 017/2014, approval date 15 April 2014) and was in accordance with the Declaration of Helsinki. The participants and their legal guardians were informed about the potential risks and benefits of the procedures involved and both provided written consent. 

### 2.2. Design

**Figure 1:**
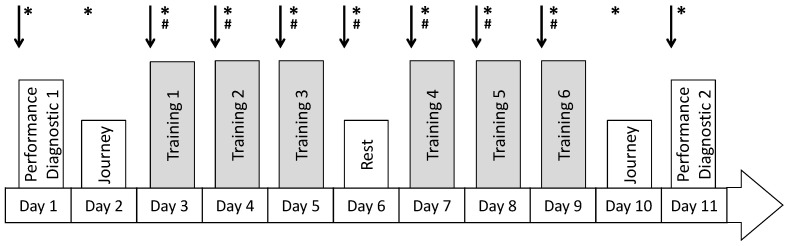
Exercise study protocol. Arrows indicate the blood sampling for the analyses of the biomarkers, asterisks (*) the request of the self-perception (*), hashtags (#) the measurement of autonomic nervous system.

The participants had all taken part in previous studies in the laboratory and were thus fully familiar with the cycling protocols and testing procedures. The focus of the training camp was the improvement of aerobic performance. The cycling training consisted of two training blocks (T1–3: Training day 1–3; T4–6: Training day 4–6), each lasting three days, separated by one resting day (R) (Figure 1).

Two days before and after the training camp, performance diagnostics (PD1, PD2) were conducted: 1. vertical counter movement jump (CMJ) for assessing lower limb explosive power (flight time was measured to calculate jump height with Optojump (Microgate, Bolzano, Italy)); 2. a graded exercise test (initial workload of 80 W and 20 W increments every 3 min until volitional exhaustion) on an electromagnetically braked cycle ergometer (SRM GmbH, Jülich, Germany) to assess oxygen uptake (VO_2_peak, Cortex Metamax^®^ 3B, Cortex Biophysik, Leipzig, Germany), maximal aerobic power (MAP) and lactate threshold (LT1 = increase in lactate > 0.4 mmol·L^−1^ [25], LT2 = fixed point at 4 mmol·L^−1^) (EKF Diagnostic Sales, Magdeburg, Germany); 3. a 30s isokinetic sprint test (isokinetic mode with a cadence of 120 rpm) to determine peak power output (PPO), mean power output (MPO), and peak lactate concentration.

During the training camp, a set of parameters were collected every morning between 7 and 9 a.m. in a fasted state: 

Training load measures: The training load was determined retrospectively using LacTRIMP (sum of the multiplications of the times spent in three different intensity zones (zone 1: ≤ LT1; zone 2: LT1—LT2; zone 3: ≥ LT2) by the coefficient relative to each zone (zone 1 = 1; zone 2 = 2; zone 3 = 3)) [26], rating of perceived exertion (RPE) for the entire session using the modified 10-point scale, and a session-RPE (s-RPE = duration of training ·RPE) [27]. Based on the intensity (mean HR and post-exercise RPE) and load (sRPE and TRIMP) measures, intensity (RPE:HR) and load (sRPE:TRIMP) ratios were calculated [11]. 

Biomarker testing: 300 µL of capillary blood from the earlobe was collected and transferred in heparinized centrifuge tube for the analyses of CK, BUN, Alb, Bil, AST, ALT, and TP (Spotchem EZ SP-4430, Arkray, Shiga, Japan; distribution: Axonlab, Baden-Dättwil, Switzerland). Serum levels were determined by optical measurement (wavelengths 550 or 610 nm) of reflection intensity of reagent color reaction on three reagent strips (AST, ALT, Bil, Alb, TP; CK; BUN). The reagent strip is composed of a multilayered test field containing reagents necessary to generate a color that is quantitated by reflectance spectrophotometry. The precision (CV %) of consecutive replicate measurements were 3.8 % for CK, 3.1 % for BUN, 2.7 % for Alb, 2.1 % for Bil, 3.5 % for AST, 1.5 % for ALT, and 3.2 % for TP. The system was checked with daily quality controls. Urine-specific gravity was measured using a digital refractometer (Index Instruments limited, Ramsey, Great Britain).

Self-perception: Person’s perceived physical state (PEPS) was assessed using psychometric questionnaires including perceived physical energy, fitness, flexibility, health [28]. Muscle soreness was assessed by sitting down on a chair from an upright posture and standing up again from this position without using the arms (10-cm visual analog scale). Self-reported sleep perception was assessed using a 1–6 scale (one being perfect). 

Autonomic nervous system: Resting heart rate (HR) and beat-to-beat R-wave to R-wave intervals (Polar 810, Polar Electro GmbH, Büttelborn, Germany) were measured every morning for 10 min in supine position upon awakening [10].

### 2.3. Statistical Analysis

Statistical analyses of the data were performed using a statistics software package IBM SPSS (version 28.0, Chicago, IL, USA). Descriptive statistics of the data are presented as means ± standard deviation (± SD). For statistical analyses, the mean of PD1 and T1 was set as baseline value (BL); however, in the graphs, both single values are shown. In case no PD1 value was available, only T1 was set as the baseline. Due to the small sample size, only the effect size (ES) Cohen’s *d*, corrected for the correlation between the BL and the respective timepoints during the training camp (T2; T3; R; T4; T5; T6; J2; PD2), are calculated [29]. Qualitative interpretation of *d* was based on the guidelines provided by Hopkins: 0 to 0.19 trivial; 0.20 to 0.59 small; 0.6 to 1.19 moderate; 1.20 to 1.99 large; 2.00 to 3.99 very large; ≥4.0 nearly perfect [30].

## 3. Results

Data of the performance tests including ES are shown in Table 1. Small increases were present for LT1 and LT2, a moderate decrease for peak lactate concentration, and a small decrease for counter movement jump height.

All athletes performed the same training sessions during the training camp. The training load measures of each day are presented in Table 2.

**Figure 2:**
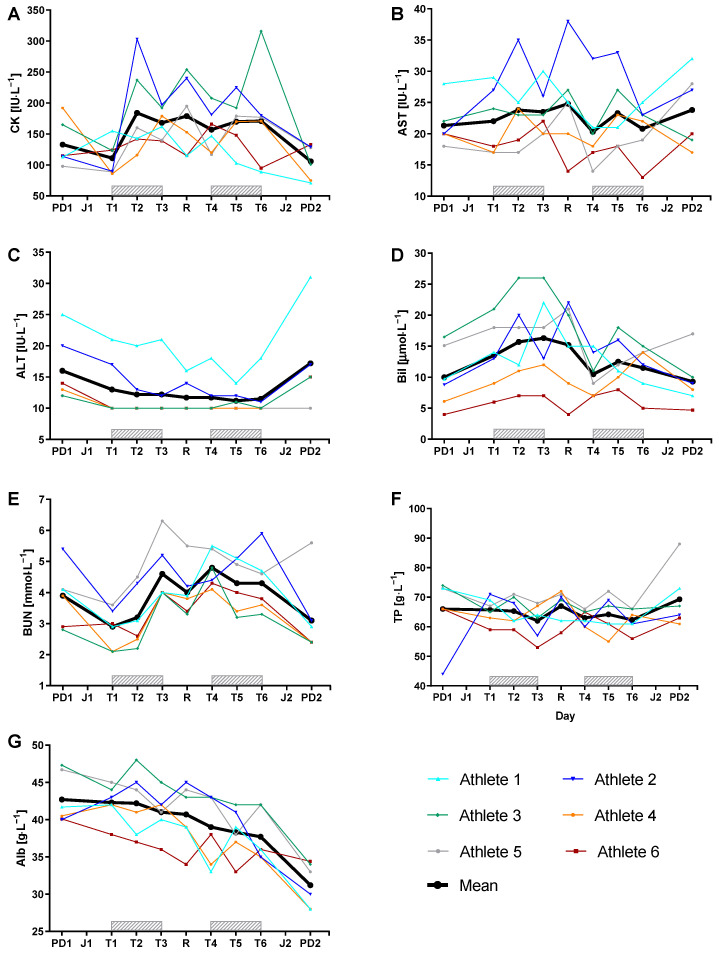
Individual day-to-day variation of different biomarkers from six young cyclists during a 7-day training camp. The black line represents the mean of all subjects. PD1/2: performance diagnostic 1/2; J1/2: journey 1/2; T1–T6: training day 1–6; R= rest day; CK: creatine kinase; AST: aspartate aminotransferase; ALT: alanine aminotransferase; Bil: bilirubin; BUN: blood urea nitrogen; TP: total protein; Alb: albumin.

Data of the biomarkers including ES are shown in Figure 2 and Table 3.

CK showed moderate to large increases during the training camp and a moderate decrease below baseline two days after the last training session. AST showed only small changes during the training camp. ALT showed trivial to small decreases during the training camp and a small increase above baseline two days after the last training session. Bil showed small to moderate increases due to the training load and small decreases after rest days. BUN showed moderate to very large increases during the training camp and a small decrease below baseline two days after the last training session. TP showed moderate decreases during the training camp and a small increase above baseline two days after the last training session, however, these changes are slightly larger than the measurement variance of the device. Alb showed moderate to large decreases during the training camp and a very large decrease below baseline two days after the last training session.

**Figure 3:**
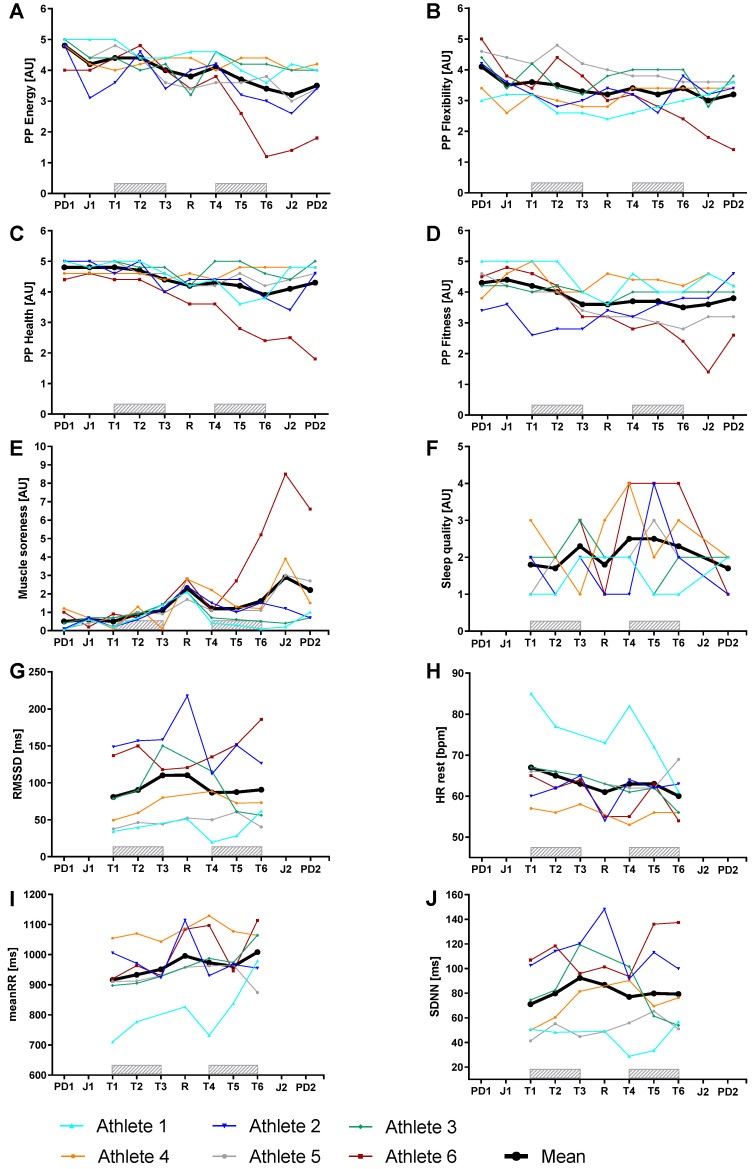
Individual day-to-day variation of the parameters of self-perception and autonomic nervous system from six young cyclists during a 7-day training camp. The black line represents the mean of all subjects. PP Energy: perceived physical energy; PP Flexibility: perceived physical flexibility; PP Health: perceived physical health; PP Fitness: perceived physical fitness; RMSSD: square root of the mean of the sum of the squares of differences; meanRR: mean R-R intervals; SDNN: standard deviation of normal R-R intervals.

Data of the self-perception and autonomic nervous system including ES are shown in Figure 3 and Table 3.

Perceived Physical Energy, Flexibility, Health, and Fitness showed moderate to large decreases during the training camp and moderate to large decreases below baseline two days after the last training session. Muscle soreness showed moderate to very large increases during the training camp and two days after the last training session compared to baseline. Sleep quality showed a moderate increase during the training camp and a small decrease below baseline two days after the last training session. Autonomic nervous system (RMSSD, HR rest, MeanRR, SDNN) showed trivial to small changes during the training camp.

## 4. Discussion

The present study aimed to investigate the external and internal training load using a multilevel approach including training load measures, biomarkers, self-perception, measures of the autonomic nervous system, and performance tests in young cyclists during a 7-day training camp. The single training load measures and the intensity and load ratios showed a similar course, therefore, in contrast to Sanders et al., the ratios did not add additional information [11]. The biomarkers CK, BUN, and Alb showed moderate to (very) large responses to the training load, and mainly returned to baseline values two days after the last training session. The self-perception showed moderate to large decreases and the measures of the autonomic nervous system (HRV) remained unchanged.

Biomarkers: According to Banfi et al. intense and continuous exercise, training and competitions can induce changes in the concentrations of numerous biomarkers [14]. In the present study, CK showed a load-dependent response with an increase after the first training day (∆ +72 ± 82 IU·L^−1^) and a decrease after the rest day (∆ −22 ± 52 IU·L^−1^) and the last training day (∆ −65 ± 86 IU·L^−1^). Generally, increases in CK during and after exercise are dependent on exercise duration, intensity, and type of muscle contraction [31,32]. Our results are in line with those from the study conducted by König et al., who also measured an increase of CK (85.3 ± 29.5 to 108 ± 36.4 IU·L^−1^) after the fourth stage of a 5-day cycling race [31]. During a training camp of distance runners with daily monitoring of biomarkers, CK values were higher (451.8 ± 138.6 IU·L^−1^) but stayed within the individual reference range [24]. The higher values in runners might be due to the higher eccentric load during running compared to cycling. Normally, eccentric exercise causes the highest CK values, but it was previously demonstrated that prolonged and fatiguing swimming exercise, an activity known to lack eccentric contractions, can induce changes in membrane permeability resulting in increased CK efflux independent from mechanically induced damage [13,33,34]. ALT showed no changes, whereas AST showed small changes in response to the training load. The time course of AST is similar to that of CK, which may be revealing the muscular demands. However, AST and ALT could also be a marker for increased demands on the liver as the main source of glycogen. In a study with elite road cyclists, AST and ALT were not abnormally elevated [35], which is in line with the data from the study conducted by Lippi et al., who pointed out that abnormalities in the liver profile based on exercise should be connected with muscle rather than liver damage [36]. BUN showed a similar load-dependent response such as CK, suggesting an enhanced muscle protein catabolism or reduced protein synthesis. This is in line with previous studies, showing an increase in BUN in professional cyclists after the fourth stage of a 5-day race [31]. The increased BUN levels in the present study are in the upper end of the clinical range (3.6–7.1 mmol·L^−1^; athletic male range 5.0–7.0 mmol·L^−1^) indicating a normal response to increased physical training [37]. An increase of BUN due to weight changes or reduced fluid intake could be excluded as in the present study no changes in body weight during the training camp were present. Furthermore, the hydration state (urine density) was checked every morning after the subject woke up before the blood markers were measured; no dehydrated status was shown. The present increase in Bil throughout the training days and the decrease after the rest day may be attributed to the continuous erythrocyte hemolysis or waste products of hemoglobin during mechanical damage during continuous muscle contractions [14]. Increased Bil levels were also shown in runners after ultraendurance races [20,38]. In the present study, TP showed only small changes during the training camp. This is in contrast to Voss et al. who showed a continuous decrease for TP during a 6-day simulated intense cycling race, which might be caused by immunological processes during an infection [39]. In contrast to TP, Alb decreased continuously from the beginning to the end of the training camp, which is in line with a study of Voss et al., also showing a continuous decrease of Alb during a 6-day simulated intense cycling race [39]. It has been shown that an increased catabolism and a decreased synthesis of albumin have the potential to cause the greatest fall in Alb concentrations [40]. The primary source for a decrease of Alb can be attributed to changes in plasma volume. Given that, TP does not follow the Alb pattern, this is most probably related to an immune response with increases in inflammation-related proteins. A factor that may lead to changes in the concentration of TP and Alb is dehydration. Therefore, some studies use correction factors to correct for dehydration based on changes in hematocrit or TP concentration [22]. As blood samples were taken in the morning, 14–16 h after the last training session, and hydration status was checked with urine state in the morning, no corrections were used in the present study.

Self-perception: The increase in training load is associated with a decrease in the psychological state (perceived physical energy, flexibility, and fitness) and sleep quality and an increase in muscle soreness. This is in line with a study of triathletes, showing a decrease in the psychological state with increased training load [41]. In general, it was noticeable that the youngest subject (athlete 6) showed a greater decrease in the perceived physical state and a greater increase in muscle soreness compared to the other athletes.

Autonomic nervous system: Resting HR and HRV remained relatively unchanged during the training camp. This result is similar to a study measuring lnRMSSD (square root of the mean of the sum of the squares of differences of successive R-R intervals) during a 1 week karate training camp, where lnRMSSD in a seated position was found to be likely higher on the first training day and remained stable during the following days [42]. However, parasympathetic hyperactivity was found in training overload studies using a longer overload time such as two weeks in runners/triathletes [43] and three weeks in trained triathletes [44]. Bellenger et al. and Le Meur et al. both reported that parasympathetic hyperactivity was accompanied by a decreased performance [43,44]. As the performance was stable in the present study, this might explain the lack of changes in resting HR and HRV.

## 5. Conclusions

The biomarkers used in the present study were sensitive to changes in the training load in young cyclists, suggesting that they are suitable to quantify the internal training load and that they can be used to control the training process. If POCT is used in training camps with young athletes, CK, BUN, and Alb in combination with psychometric questionnaires seem to be the best parameters to show changes in training load, which may allow the individual regulation of training load.

## Figures and Tables

**Table 1:** Changes in physical performance before and after the training camp (mean ± SD). MAP: maximal aerobic power; VO_2_peak: peak oxygen uptake; LT1/2: lactate threshold 1/2; PPO: peak power output; MPO: mean power output.

	**Pre**	**Post**	**Mean Difference**	**ES**
**Graded Exercise Test**				
MAP [W]	241 ± 26	246 ± 27	4 ± 9	0.18
VO_2_peak [mL·min^−1^·kg^−1^]	57.8 ± 4.5	59.2 ± 6.9	1.4 ± 3.7	0.19
LT1 [W]	140 ± 22	150 ± 21	10 ± 11	0.46
LT 2 [W]	201 ± 22	209 ± 20	7 ± 9	0.33
**Wingate Anaerobic Test**				
PPO [W]	928 ± 193	921 ± 186	−6 ± 55	−0.03
MPO [W]	641 ± 69	623 ± 107	−23 ± 44	−0.15
Peak lactate concentration [mmol·L^−1^]	10.6 ± 1.5	8.9 ± 1.4	1.7 ± 0.8	−1.08
**Counter Movement Jump**				
Height [cm]	29.9 ± 4.1	28.6 ± 3.5	−1.3 ± 2.2	−0.33

**Table 2:** Training load measures for each variable and each day of all athletes (mean ± SD). T1–6: training days 1–6; R: rest day; HR: heart rate; RPE: rating of perceived exertion, sRPE: session-RPE; TRIMP: training impulse.

	T 1	T 2	T 3	R	T 4	T 5	T 6
Duration [min]	120	250	255		170	300	180
Distance [km]	52	103	95		73	122	72
Altitude difference [m]	700	925	1300		610	1335	950
Mean HR [bpm]	146 ± 11	139 ± 8	129 ± 8		133 ± 9	132 ± 9	135 ± 11
Mean HR % HR_max_	74 ± 2	71 ± 3	66 ± 2		68 ± 3	67 ± 3	69 ± 2
HR_max_ [bpm]	186 ± 19	191 ± 19	185 ± 11		180 ± 6	175 ± 10	180 ± 10
RPE [AU]	4 ± 2	5 ± 1	7 ± 1		6 ± 2	7 ± 1	6 ± 1
sRPE [AU]	528 ± 218	1367 ± 207	1883 ± 353		963 ± 372	2115 ± 445	1152 ± 373
LacTRIMP [AU]	151 ± 29	289 ± 39	293 ± 27		189 ± 21	327 ± 51	215 ± 38
Intensity ratio: RPE:HR [AU]	2.91 ± 1.63	3.45 ± 0.58	5.74 ± 1.02		4.81 ± 1.32	5.36 ± 1.13	4.98 ± 1.39
Load ratio: sRPE:TRIMP [AU]	3.34 ± 1.53	4.78 ± 0.86	6.42 ± 0.95		5.72 ± 1.29	6.47 ± 1.05	5.66 ± 1.32

**Table 3:** Effect sizes (Cohen’s *d*) for the biomarkers, self-perception, autonomic nervous system related to baseline values. BL: Mean of PD1 & T1 (only T1); T2–T6: training day 2–6; R: rest day; J2: journey 2; PD2: performance diagnostic 2; CK: creatine kinase; AST: aspartate aminotransferase; ALT: alanine aminotransferase; Bil: Bilirubin; BUN: blood urea nitrogen; TP: total protein; Alb: albumin; PP: perceived physical; RMSSD: square root of the mean of the sum of the squares of differences; meanRR: mean R-R intervals; SDNN: standard deviation of normal R-R intervals.

	**BL-T2**	**BL-T3**	**BL-R**	**BL-T4**	**BL-T5**	**BL-T6**	**BL-J2**	**BL-PD2**
**Biomarkers**								
CK	1.23	1.99	1.28	1.15	1.49	0.76		−0.66
AST	0.33	0.35	0.41	−0.30	0.26	−0.27		0.34
ALT	−0.45	−0.50	−0.11	−0.48	−0.33	−0.58		0.34
BUN	0.54	0.62	0.45	−0.33	0.10	−0.10		−0.55
TP	−0.16	1.34	0.84	2.12	1.05	0.74		−0.23
Alb	−0.17	−0.75	0.15	−0.71	−0.35	−0.83		0.35
**Self-Perception**								
PP Energy	−0.53	−1.46	−1.51	−1.13	−1.52	−1.12	−1.28	−1.30
PP Flexibility	−0.36	−0.95	−1.01	−0.88	−1.06	−0.82	−1.39	−0.86
PP Health	−0.27	−1.28	−2.00	−1.10	−0.79	−1.10	−0.80	−0.34
PP Fitness	−0.27	−1.06	−1.04	−0.74	−0.89	−0.98	−0.64	−0.60
Muscle soreness	1.37	1.41	4.70	1.34	0.70	0.50	0.47	0.59
Sleep quality	−0.25	0.64	0.00	0.62	0.60	0.53	1.52	−0.26
**Autonomic Nervous System**								
RMSSD	0.16	0.40	0.27	0.12	0.13	0.18		
HR rest	−0.14	0.10	−0.77	−0.35	−0.29	−0.86		
MeanRR	0.12	−0.10	0.84	0.42	0.31	0.88		
SDNN	0.28	0.57	0.23	0.21	0.24	0.25		

## Data Availability

The data presented in this study are available on request from the corresponding author.
